# The Progress of Non-Viral Materials and Methods for Gene Delivery to Skeletal Muscle

**DOI:** 10.3390/pharmaceutics14112428

**Published:** 2022-11-10

**Authors:** Zhanpeng Cui, Yang Jiao, Linyu Pu, James Zhenggui Tang, Gang Wang

**Affiliations:** 1 National Engineering Research Center for Biomaterials, College of Biomedical Engineering, Sichuan University, Chengdu 610064, China; 2 School of Materials and Chemistry, Southwest University of Science and Technology, Mianyang 621010, China; 3 Research Institute in Healthcare Science, Faculty of Science & Engineering, University of Wolverhampton, Wolverhampton WV1 1SB, UK

**Keywords:** non-viral materials, gene delivery, skeletal muscle, polymer

## Abstract

Since Jon A. Wolff found skeletal muscle cells being able to express foreign genes and Russell J. Mumper increased the gene transfection efficiency into the myocytes by adding polymers, skeletal muscles have become a potential gene delivery and expression target. Different methods have been developing to deliver transgene into skeletal muscles. Among them, viral vectors may achieve potent gene delivery efficiency. However, the potential for triggering biosafety risks limited their clinical applications. Therefore, non-viral biomaterial-mediated methods with reliable biocompatibility are promising tools for intramuscular gene delivery in situ. In recent years, a series of advanced non-viral gene delivery materials and related methods have been reported, such as polymers, liposomes, cell penetrating peptides, as well as physical delivery methods. In this review, we summarized the research progresses and challenges in non-viral intramuscular gene delivery materials and related methods, focusing on the achievements and future directions of polymers.

## 1. Introduction

Due to the wide distribution and enormous number of skeletal muscles in the human body, it has high application value to deliver functional genes into skeletal muscle cells for target protein expression. Skeletal muscles can act as protein factories, wherein exogenous functional genes can serve as “blueprints” to produce target proteins for specific purposes, such as antibodies for cancer immunotherapy [1] and insulin analogues for diabetes treatment [2]. Delivery of exogenous genes into cells needs vectors, which are mainly divided into viral vectors and non-viral vectors. Although viral vectors had high gene delivery efficiency, their potential biosafety risks limited the clinical applications [3]. In 1990, Jon A. Wolff [4] first reported that skeletal muscles could take up plasmid DNA (pDNA) and express the reporter protein. In the experiments, naked pDNA carrying LacZ gene was directly injected into a quadriceps muscle of mice and seven days later, the whole muscle was removed and stained by X-Gal. The stained cross-section of the muscle tissue showed that the target gene could be expressed in muscle cells to a certain extent. However, it is difficult to acquire high gene delivery efficiency and expression level via direct injection of naked pDNA into skeletal muscles.

**Scheme 1:**
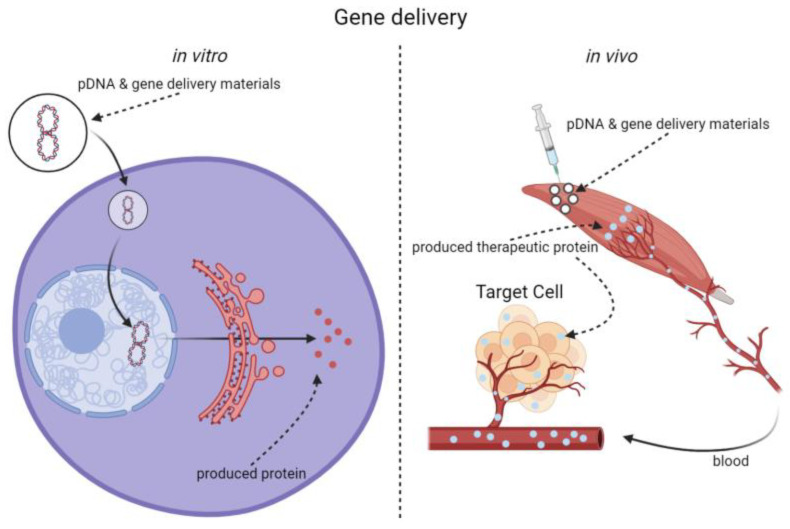
Differences between gene delivery in vivo and transfection in vitro.

Transfection of pDNA into cultured cells is a process in which cells actively or passively acquire foreign DNA in a simple in vitro environment. The main barriers of gene transfection include cell plasma membrane, escape of genes from endosomes and lysosomes, detachment of genes from gene carriers, nuclease, and cell nuclear membrane, etc. [5,6]. In comparison, delivery of pDNA into skeletal muscle cells is carried out in a more complex in vivo environment, so it is confronted with extracellular and intracellular obstacles. Before penetrating the cell plasma membrane, it needs to overcome the extracellular obstacles first. Owing to the large size and negative charge of pDNA molecules, naked pDNAs can be easily trapped or damaged by extracellular obstacles, such as extracellular matrix (ECM), biomacromolecules with positive charge as well as nuclease in the ECM [5] (Scheme 1). To solve this problem, Russell et al. combined polyvinyl pyrrolidone (PVP, Table 1) and polyvinyl alcohol (PVA) with pDNA in mixed solution and injected the solution into the tibialis anterior (TA) muscles, generating improved expression level of exogenous genes compared to naked pDNA injection [7]. The study showed that gene delivery efficiency into skeletal muscles could be improved by some non-viral biomaterials.

In contrast to the prosperity of non-viral vectors regarding in vitro gene transfection of cells and in vivo targeted gene delivery through the bloodstream, there were limited studies on non-viral vectors and related methods for skeletal muscle gene delivery [28]. The following points should be cautiously considered when carrying out intramuscular gene delivery: (1) preventing DNAs from being trapped in ECM; (2) protecting genes from being degraded by nucleases in ECM; (3) facilitating genes to penetrate cytoplasmic membranes and enter nuclei of target cells; (4) having biosafety without any side effects on living organisms.

## 2. Advantages and Challenges of the Skeletal Muscle Gene Delivery

For the traditional gene therapy, the therapeutic genes were usually sent to the lesion cells, such as in cancer treatment [29,30,31]. However, for the skeletal muscle-based gene therapy, the delivery targets were skeletal muscle cells. Previous studies have screened the muscle-specific promoter [32], proving that skeletal muscle has microbubbles, which can bring the protein secreted out of the cells, affect the adjacent cells, and regulate the behaviour of them [33]. In addition, proteins secreted by muscle cells can also enter the circulatory system and affect physiological parameters, such as hormone secretion, and nervous and immune system activity [34]. Based on the above points, some scientists have successfully introduced the vector carrying insulin gene into skeletal muscle with genetic engineering technology and used it to treat Type 1 diabetes [35]. After that, another study showed that the co-expression of insulin and glucokinase could be applied to correct hyperglycaemia and prevent hypoglycaemia [36]. It can be concluded that in these systems, skeletal muscle cells played the role as protein factories, rather than a lesion site.

### 2.1. Advantages of the Skeletal Muscle as the Target for Gene Delivery

Skeletal muscles have many inherent anatomical, cellular, and physiological characteristics. It is a good target tissue for gene expression, especially for the production of proteins such as systemic therapeutic agents. The significant advantages of skeletal muscle as a target tissue for gene delivery can be mainly attributed to four points:

(1) The weight of skeletal muscles accounts for 30% of a normal adult’s weight, which means there is enough target tissue for gene delivery; (2) Skeletal muscles have rich vascular systems. Abundant capillary networks wrap each muscle fiber at regular intervals, thus providing an effective transportation system for secreted proteins to enter the circulation; (3) Skeletal muscle fibers contain terminally differentiated cells, and the nuclei in the fibers are postmitotic. A single muscle fiber can survive in the living body for a long time. Even if muscle fibers are damaged and only a short segment of some fibers is unimpaired, the nucleus of surviving muscle can still work [37]. It provides a stable environment for the continuous production of proteins; (4) In muscle fibers, foreign genes can be spread from a limited injection site to the nuclei of a large number of adjacent muscle cells in the fiber. This ability to diffuse foreign genes in muscle fibers is probably one of the reasons why foreign genes can be highly expressed in muscles [28].

Therefore, skeletal muscles are expected to become a potential target tissue for gene therapy. Gene delivery to skeletal muscle is simple as it only requires intramuscular injection, differing from long-term daily insulin injection or radiotherapy and chemotherapy.

### 2.2. Obstacles to Skeletal Muscles Gene Delivery

Systemic administration of genes transports foreign vectors through the circulatory system, which may face obstacles of off-target effects on other organs and stability changes in serum. As a local injection method, gene delivery to skeletal muscle cells allows direct injection of prepared plasmid DNA. Therefore, the obstacles mainly exist in the ECM, cytoplasmic membrane, endosome escape, and nuclear entry.

#### 2.2.1. Obstructions in the Extracellular Matrix

Unlike other ECMs, the ECM of the skeletal muscle is a three-dimensional scaffold composed of various collagens, glycoproteins, proteoglycans, and elastin [38], and many of these proteins are negatively charged [39].

Generally, the delivery efficiency of most gene carriers, such as liposomes and polymers, is much lower than that of viral vectors. Unlike other organs, muscle fibers are surrounded by a mechanically strong ECM, which is the glycosaminoglycan-rich basement membrane in the skeletal muscle [40,41,42] and the ECM is strongly negatively charged. As a result, cationic liposomes and polymers are very easily bound to the negatively charged biomacromolecules in the ECM, which hinders the entry of cationic DNA/carriers complexes into cells [43].

Naked plasmids have shown to be useful for gene transfer into skeletal muscles [4], but low transfer efficiency brought great challenges to clinical promotion. Studies have shown that adding nuclease inhibitors could improve the transfer efficiency of pDNA into muscles [44], which proved that nuclease was also one of the main factors in ECM that inhibited pDNA transfer efficiency.

#### 2.2.2. Cytoplasmic Membrane

The existence of the cell membrane provides a relatively stable environment for the cell. The main structure of the cytoplasmic membrane is a phospholipid bilayer containing phospholipid molecules, cholesterol, and proteins embedded in the membrane or on the membrane surface. Highly hydrophilic and bulky pDNA molecules are easily blocked due to the existence of the amphiphilic bilayers in the cytoplasmic membrane. Therefore, how the gene and the carrier cooperate to cross the cytoplasmic membrane and enter the cell is also a key issue in gene delivery. So far, the methods used to improve the delivery efficiency of pDNA mainly included adding components that could promote the internalization of DNA into target cells, such as transferrin [45], organic solvents, nonionic polymers or surfactants, etc. [5]. According to the properties of these substances, these additives were speculated to temporarily change the permeability of the cytoplasmic membrane by disturbing the cytoplasmic membrane, and therefore allowing pDNA to penetrate the membrane more easily [46]. In addition to adding the above substances, some physical methods could temporarily create non-lethal pores in the cell membrane to facilitate the passage of pDNA, such as gene gun [47], electroporation [48], ultrasonic microbubble [49], and hydrodynamic [50] methods.

#### 2.2.3. Endosomal Escaping

For osmotic and invasive delivery methods, genes generally enter the cytoplasm directly through the cytoplasmic membrane and do not involve endosomal escape. The delivery method of genes into cells by endocytosis needs to consider the problem of endosomal escape. After genes undergoing endocytosis, endosomes/lysosomes may form, and lysosomes will decompose foreign substances into small molecules for reuse, which will make the therapeutic gene ineffective. Therefore, target genes should escape from endosomes after endocytosis to avoid being degraded.

Generally, cationic polymers mainly escape from endosomes to mediate gene delivery, which is represented by branched polyethyleneimine (bPEI, 25 kb) [51]. The bPEI may form PEI-DNA complexes first, enter cells through endocytosis, then escape from the endosome into the cytoplasm through the “proton sponge” effect [52].

#### 2.2.4. Entering the Nucleus

For viral vectors, the virus can accomplish the processes of membrane entry, endosomal escape, and entry into the nucleus only by its shell. The delivery efficiency of non-viral gene delivery methods is generally low. The reason may be that the functionality of non-viral gene delivery materials is not complete. In general, numerous studies of non-viral delivery methods have focused on cytoplasmic membrane penetration and endosomal escape. However, in the whole process of gene delivery, whether the exogenous gene can enter the nucleus for expression stably is one of the key factors affecting the efficiency of gene expression. The nucleus is the centre of the cell surrounded by two membranes, and few small particles can freely pass in and out through the nuclear pores [6].

For dividing cells, gene delivery becomes relatively easy, because during cell division the nuclear envelope breaks, making it easier for foreign genes to enter the nucleus [6]. Studies have shown that low molecular weight bPEI/DNA complexes enter the nucleus more easily than the high molecular weight bPEI/DNA complexes [53]. But for cells without division, gene delivery becomes relatively complicated and difficult, and the nuclear membrane blocks larger-sized molecules, which is one of the major challenges of gene delivery.

For this problem, nuclear localization signal (NLS) peptides [54] were used, which can help larger particles to complete the nuclear entry by embedding nuclear pores in the nuclear envelope. Subsequent studies have shown that binding to NLS polypeptides could improve gene entry and expression in non-dividing cells [6,55].

#### 2.2.5. Material Stability

Skeletal muscle gene delivery requires sufficient material stability in the ECM, and then easily to be degraded or cleared in cells. For cationic gene delivery materials, it is easy to form cationic material/DNA complex, but the cationic complex can electrostatically interact with negatively charged biomacromolecules in the ECM, leading to significantly reduced gene delivery efficiency. Meanwhile, the strong binding of material molecules to DNA does not necessarily improve the transfection efficiency, since it may hinder the unpacking of the cationic material and DNA [56]. For branched PEI, the representative of cationic polymers, high molecular weight polymers can bind to DNA better to protect DNA from degradation, and are more readily taken up by cells, resulting in higher delivery efficiency, but low molecular weight polymers have lower cytotoxicity and better DNA unpacking ability [57,58]. Therefore, it can be speculated that there should be an optimal molecular weight range of polymers, so that these polymers have suitable DNA compression and unpacking ability with better biosafety and gene delivery efficiency as well.

#### 2.2.6. Biosecurity

Although there are many methods with high gene delivery efficiency, there are also biological safety issues, such as cytotoxicity, inflammatory response, and immune reaction. For in vivo gene delivery, biosafety is an unavoidable issue. Although viral vectors have high gene delivery efficiency and expression level, they can easily trigger excessive inflammatory responses [59] or immune reaction, while non-viral delivery methods avoid the occurrence of side effects to a great extent. For cationic polymers, the toxicity mainly comes from excessive positive charges. At present, polyethylene glycol (PEG) has been used to shield the cations on the surface of the complex to reduce cytotoxicity [60]. Grandinetti et al. showed that the polymer could directly participate in the nuclear localization of DNA through its membrane destruction characteristics, which may also be the reason for its cytotoxicity [61]. Therefore, it seems to be a better choice to use neutral block polymers since they are more effective than cationic polymers in skeletal muscle gene delivery [14,18,19,62].

## 3. Biomaterials for Skeletal Muscles Gene Delivery

### 3.1. Polymers

**Figure 1:**
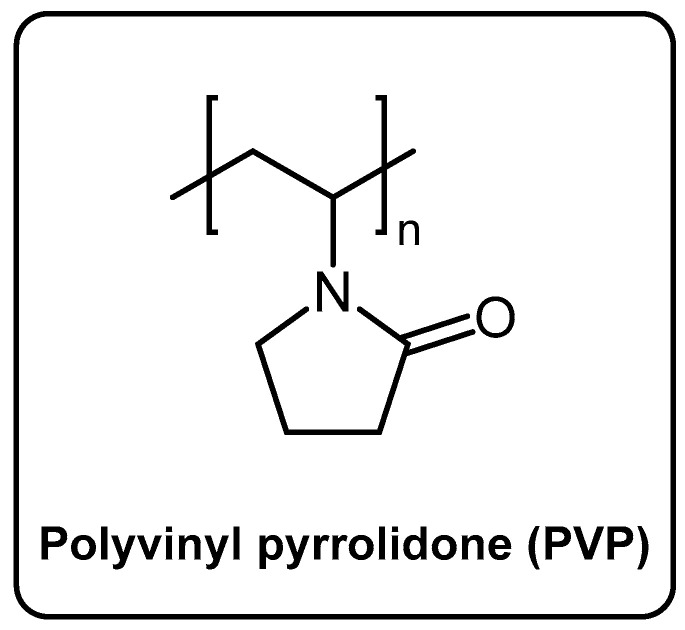
The chemical structure of PVP.

As early as 1996, Russell et al. reported the application of polyvinylpyrrolidone (PVP, Figure 1) for intramuscular delivery of the plasmids CMV-CAT or CMV-β-CAT into the tibial muscle of rats [7]. The authors used an isotonic solution containing 5% PVP as a carrier for delivering the pDNAs to the skeletal muscle, and acquired better results in comparison with the naked plasmid injection, but the gene expression levels were still limited. Since this report, more materials have been developed for gene delivery to skeletal muscles.

#### 3.1.1. Block Co-Polymers

(1) Triblock co-polymers

- Pluronic^®^ block co-polymers

**Figure 2:**
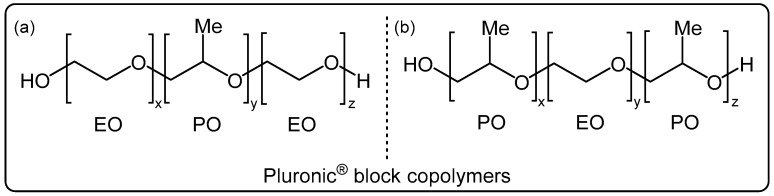
The chemical structure of Pluronic^®^ triblock copolymer. (PO: Propylene oxide, EO: Ethylene oxide). (**a**) This structure contain Pluronic^®^ L64, P85, L61, F127, P105 and SP1017, (**b**) This structure contain Pluronic^®^ 25R2 and 25R4.

Pluronic^®^ triblock co-polymer is a pharmaceutical excipient recognized by the United States and British Pharmacopoeia. It has been widely used in the research of gene delivery. The structure of the Pluronic^®^ block copolymer is shown in Figure 2, which is composed of block poly propylene oxide (PEO), block poly methyl propylene oxide (PPO), and block poly propylene oxide (PEO). Pluronic^®^ Block copolymer is an amphiphilic and neutral polymer, which can form micelles by self-assembly in aqueous solutions. It is a dispersion when the concentration of the block copolymer in water is lower than the critical micellar concentration (CMC) and the macromolecules are molecularly dissolved, while above CMC they aggregate and form micelles through a self-assembly process. The hydrophobic PO blocks can form cavities inside the micelles, which can carry drugs and genes, enabling gene delivery [63].

- Pluronic^®^ P85

In subsequent studies, researchers found that Pluronic^®^ P85 (PEO_26_-PPO_40_-PEO_26_, Figure 2a, Table 1) had the ability to deliver pDNAs to skeletal muscles.

In 2009, Gaymalov et al. injected a mixed solution of P85 and pDNA encoding GFP or Luc into the thigh skeletal muscle of mice [9]. The gene expression not only increased in the muscle, but also significantly increased in the spleen, inguinal lymph nodes, and liver, which provided a possibility to treat cancer, diabetes and other diseases by delivering therapeutic pDNA to the muscle. In 2016, Mahajan et al. continued to report the application of P85 in gene delivery [10]. The author established a murine hind limb ischemia model (MHLIM) in mice. It was found that the efficiency of P85 in delivering DNA could be enhanced in the presence of local inflammation in the hindlimbs. Inflammation may lead to an increasing count of neutrophils in blood neutrophils and secretion of proinflammatory cytokines, such as TNF-α and IL-1 β [64]. The overall activation of inflammatory signals can affect the gene expression driven by the CMV promoter, which was manifested in the increased gene expression in MHLIM.

In 2009, Namgung et al. used P85 and di-(ethylene glycol) divinyl ether (DEGDVE, Table 1) to prepare multi-block copolymers (MBCPs) hydrogels as carriers for intramuscular gene delivery [11]. It could continuously and controllably release pDNA. The polymer had good fluidity when the temperature was below body temperature. After in vivo injection, due to the rise of temperature, it quickly changed into a gel state in situ, and could slowly release DNA locally for a long time. The author believed that after degradation of MBCPs gel, the generated MBCPs fragments would form micelles and combine with pDNA to form polyplexes less than 100 nm, thus effectively carrying pDNA into cells. The polyplexes delivered pDNA by continuously increasing the effective concentration of pDNA around a specific point, thereby increasing the internalization probability of pDNA. In the in vivo experiment, when the mixture of MBCPs and pDNA was injected into the skeletal muscle of the hind limb of mice, the MBCPs/pDNA mixture showed a significant enhancement of gene expression compared with that of naked pDNA. It was explained that Pluronics^®^ could be used as an adjuvant to activate NF-κB cell signalling to increase pDNA uptake, and to increase the efficiency of pDNA entry into the nucleus [8,14].

b. Pluronic^®^ SP1017

In 2005, Yang et al. used different concentrations of Pluronic^®^ P85 and Pluronic^®^ SP1017 (Pluronic^®^SP1017 is produced by combining Pluronic^®^ L61 with Pluronic^®^ F127, y = 31, x = z = 4.5 for Pluronic^®^ L61, and y = 65.2, x = z = 200.4 for Pluronic^®^ F127 [65], Figure 2a, Table 1) to compare the delivery efficiencies of pDNAs into the TA muscles of BALB/c mice [8]. It was found that the delivery efficiency of naked plasmid was extremely low during the whole process. When P85 was at 0.3% (*w*/*v*), the gene delivery efficiency was the highest, which was significantly higher than that of SP1017. The author used P85 as the carrier to study the time of gene delivery and expression, and found the peak was on the seventh day. Among other Pluronic^®^ compounds, Lemieux et al. reported that Pluronic^®^ SP1017 was used as a carrier for gene delivery, and SP1017 had better thermal stability and lower cytotoxicity [65]. SP1017 showed 10 times higher delivery efficiency than naked DNA. Compared with PVP, both high molecular weight PVP and low molecular weight PVP have higher skeletal muscle delivery efficiency.

c. Pluronic^®^ 25R2

In 2011, Guiraud et al. [12] reported the use of Pluronic^®^ 25R2 (PO_21_-EO_14_-PO_21_, Figure 2b, Table 1) and Pluronic^®^ 25R4 (PO_19_-EO_33_-PO_19_, Figure 2b) for skeletal muscle gene delivery. 25R2 and 25R4 showed poor performance in vitro transfection experiments in HEK 293T cells. When delivering genes to the tibialis anterior muscle, researchers used the plasmid DNA which was encoded by Luc. 25R2 shows similar delivery efficiency to P105 (PEO_37_-PPO_56_-PEO_37_ [66], Figure 2a) and L64. However, the efficiency of 25R4 is obviously lower than that of 25R2.

d. Pluronic^®^ L64

In 2002, Bruno et al. reported that they used Pluronic^®^ PE6400 [67] (Trade Name: Pluronic^®^ L64, PEO_13_-PPO_30_-PEO_13_, Figure 2a, Table 1) neutral polymer mixed with pDNA to deliver genes to skeletal muscles [13]. The pDNAs encoded luciferase (Luc) or β-galactosidase (β-Gal) as reporter genes, respectively. Compared with the naked plasmid group, the experimental group using PE6400 as the carrier had a certain improvement on reporter gene expression.

In 2014, our research team reported the application of Pluronic L64 to intramuscularly deliver pDNA encoding hypoxia inducible factor-1α (HIF-1α) for the treatment of severe hind limb ischemia in mice [68]. We also found that Pluronic L64 had better gene delivery efficiency and was more stable than P85. 

In 2015, our research team further studied the gene delivery mechanism of Pluronic^®^ L64 [46]. As an amphiphilic triblock copolymer, Pluronic^®^ L64 could interact with the phospholipid bilayer in biomembranes. The interaction could disturb the membrane structure, permeability, and endocytic function in a time and concentration dependent manner, facilitating directly pDNA transmembrane rather than endocytosis into cells. It also accelerated the unpacking of pDNA from pDNA/PEI complex and escape of pDNA from endosomes/lysosomes.

B. Other triblock co-polymers

Polyethylene glycol (PEG) modified liposomes could reduce cytotoxicity, prolong the half-life, and increase long cycle time, but they could reduce cell transfection efficiency [69]. Similarly, the polymer modified with PEG also had a similar effect in the same way.

**Figure 3:**
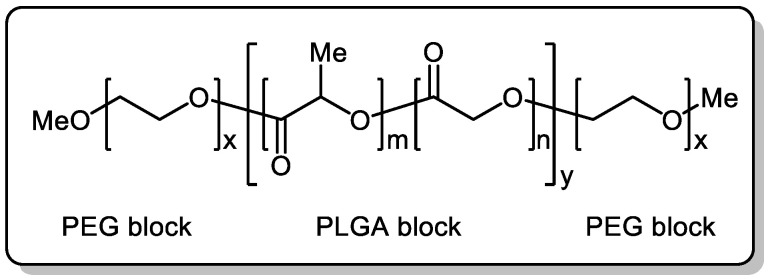
The chemical structure of PEG–PLGA–PEG triblock copolymer. For PEG_13_–PLGA_10_–PEG_13_, x = 13, y = 10, m = 3, n = 1.

In 2007, Chang et al. prepared a block polymer, PEG_13_-PLGA_10_-PEG_13_, composed of PEG (Polyethylene glycol) and poly(lactic-*co*-glycolic acid) (PLGA), which was biodegradable and therefore had low cytotoxicity [16]. In the pCMV-Luc delivery experiment to the skeletal muscle of SD rats, the luciferase activity in the group using 0.25% PEG_13_-PLGA_10_-PEG_13_ (Figure 3, Table 1) was three orders of magnitude higher than that of the bPEI (25 kDa) group, three times that of the naked plasmid group, and the PEG_13_-PLGA_10_-PEG_13_ expression peaked on the fifth day. The authors also used PEG_13_-PLGA_10_-PEG_13_ to deliver the therapeutic gene vascular endothelial growth factor (VEGF) to the tibialis anterior muscle of rats. Two days after intramuscular injection, the VEGF expression level delivered by 0.25% PEG_13_-PLGA_10_-PEG_13_ was about two times higher than that of naked pDNA. Ten days after intramuscular injection, VEGF expression levels of PEG_13_-PLGA_10_-PEG_13_/pDNA and naked pDNA were similar. The authors also studied the distribution of injected pDNA by fluorescent ethidium monoazide (EMA) labelled pDNA. From the distribution study, pDNA delivered by bPEI (25 kDa) was limited to the injection site. In contrast, the distribution of pDNA delivered by 0.25% PEG_13_-PLGA_10_-PEG_13_ could be detected not only at the injection site but also in the surrounding muscle fibers. Compared with bPEI (25 kDa)/DNA, the greater diffusion of PEG_13_-PLGA_10_-PEG_13_/pDNA may explain the better transfection efficiency in vivo.

**Figure 4:**
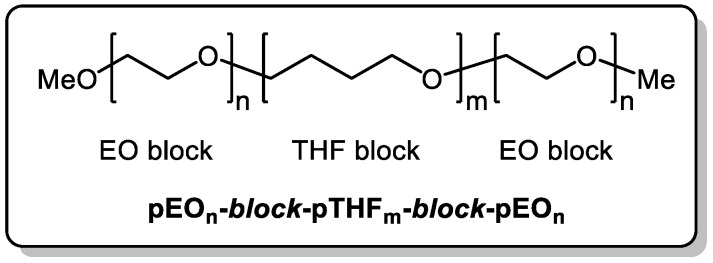
The chemical structure of pEO_n_-*block*-pTHF_m_-*block*-pEO_m_ (CP1 and CP2) triblock copolymer. For CP1 n = 15, n = 33; CP2 n = 11, m = 9.

In 2008, Pomel et al. prepared an amphiphilic poly(tetrahydrofuran-b-ethylene oxide) (PEO–PTHF–PEO, Table 1) triblock copolymer for the study of gene delivery in mouse skeletal muscles [17]. Two copolymers, CP1 (Figure 4) and CP2 (Figure 4), with different polymerization degrees were prepared for the pDNA delivery into mouse skeletal muscle. The expression of luciferase in CP1 and CP2 groups was significantly increased compared with the naked pDNA group, and the expression of luciferase was increased by 10 times one week after injection. They also repeated the experiment using another reporter gene lacZ and acquired similar results.

**Figure 5:**
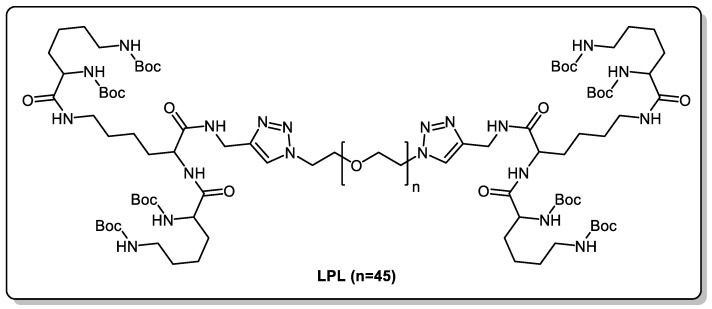
The chemical structure of LPL (n = 45) triblock copolymer.

In 2014, our research group reported the preparation of triblock copolymer dendron G2(L-lysine-Boc)-PEG_2k_-dendron G2(L-lysine-Boc) (LPL, Figure 5, Table 1) [18]. LPL was an electrically neutral copolymer and showed hydrophobic–hydrophilic–hydrophobic structure, which was opposite to that of the phospholipid bilayer of cell membranes. The LPL hydrophilic region was speculated to interact first with the outer hydrophilic side of the cell membrane. Thereafter, the flowable membrane component could be extruded by the LPL copolymer, and the internal hydrophobic fatty acid chains were exposed, followed by the entry of the hydrophobic portion of the copolymer. When the hydrophilic region of the copolymer was pushed by the hydrophobic fatty acid chain, the copolymer molecule could flip, bringing its hydrophilic region closer to the inside of the plasma membrane, thereby perturbing the cell membrane. In the qualitative assay using the plasmid encoding lacZ gene to the TA muscles of mice, the staining effects of LPL and Pluronic^®^ L64 groups were stronger than that of the naked plasmid group. In the quantitative assay using the plasmid encoding luciferase, the Luc expression in the LPL group was 17 times that of the naked plasmid group and 2.5 times that of the L64 group. Visible transgene expression in living animals is considered to be more convenient for assessing expression intensity and duration. The expression of the far-red fluorescent protein E2-Crimson was observed on days 7 and 14 after a single injection. There was no significant difference in fluorescence intensity between the pDNA/L64 (0.1%) and pDNA/LPL (0.0075%) groups, but the results of 0.0075% LPL were more stable than those of 0.1% L64, because all six sites in the pDNA/LPL (0.0075%) group still showed obvious fluorescence on day 14, while one site in the pDNA/L64 (0.1%) group lost detectable signals. In the naked pDNA group, only two of the six sites showed visible but weak fluorescence signals compared with the other two groups. Meanwhile, the fluorescence signal duration of the pDNA/LPL (0.0075%) group lasted at least 14 days without any attenuation. Further, we used plasmid expressing mouse growth hormone (mGH), which has physiological functions for mouse growth. Upon single administration of 50 μg pDNA-mGH), mice in the pDNA/LPL (0.0075%) group showed an accelerated growth rate in the first 10 days compared with those in the saline group. It demonstrated that the expression amount of mGH in pDNA/LPL (0.0075%) mice was high enough for physiological function. It can also be concluded that if a suitable therapeutic gene is used here instead of mGH, a therapeutic effect may be acquired.

**Figure 6:**
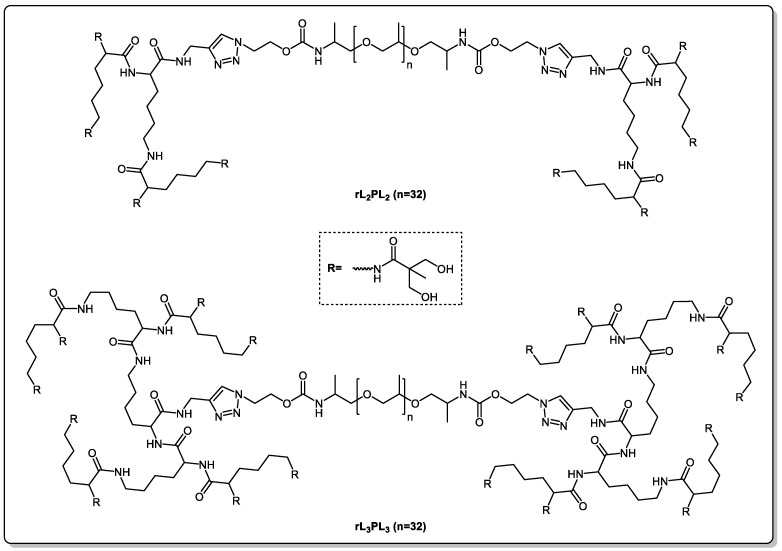
The chemical structure of rL_2_PL_2_& rL_3_PL_3_ (n = 45) triblock copolymer.

In 2016, our research group developed dendronG2(L-lysine-OH)-poly propylene glycol_2k_(PPG_2k_)-dendronG2(L-lysine-OH) (rL_2_PL_2_, Figure 6, Table 1) and dendronG3(L-lysine-OH)-PPG_2k_-dendronG3(L-lysine-OH) (rL_3_PL_3_, Figure 6, Table 1) [19] based on previous work [18]. The hydrophilicity and hydrophobicity of rL_2_PL_2_ and rL_3_PL_3_ are opposite to that of LPL, and structurally similar to that of cell membrane phospholipid bilayer. The Lac-Z expression levels mediated by L64, rL_2_PL_2_, and rL_3_PL_3_ were higher than that of naked plasmid. In the luciferase expression assays, when the mass ratio of rL_2_PL_2_ increased, the luciferase activity in the rL_2_PL_2_/pDNA group rose sharply. It peaked at 0.005% and quantitatively increased transgene expression levels 15-fold at 0.005% rL_2_PL_2_ and 2.7-fold at 0.1% L64 compared to naked DNA. For rL_3_PL_3_, although luciferase expression was improved at a concentration of 0.02%, the results fluctuated significantly, indicating that rL_3_PL_3_ mediated transgene expression was unstable. Expression levels of E2-Crimson were examined on days 7 and 14 after a single injection. By comparing the intensity and stability of the fluorescence signals in the four groups, the effect trend can be summarized as: pDNA/rL_2_PL_2_(0.005%) > pDNA/L64(0.1%) > pDNA/rL_3_PL_3_(0.02%) > naked DNA. The signal in the pDNA/rL_2_PL_2_ (0.005%) group was strong and persisted for at least 14 days without significant attenuation, indicating that rL_2_PL_2_ was a potential material that could deliver genes to skeletal muscles for therapeutic applications. In the functional gene expression assay, a single injection of 50 μg pDNA-GHRH/rL_2_PL_2_ (0.005%) showed an accelerated growth rate for 21 days. Compared with naked DNA and pDNA-GHRH/L64 (0.1%) groups, the pDNA-GHRH/rL_2_PL_2_ group showed significant growth promotion in mice weights. However, there was no significant increase in the pDNA-GHRH/rL_3_PL_3_ (0.02%) group. The results indicated that the expression of GHRH could produce physiological effects after pDNA-GHRH/rL_2_PL_2_ (0.005%) injection. It was also conceivable that some therapeutic effects might be obtained if the appropriate therapeutic gene was used here instead of GHRH.

**Figure 7:**
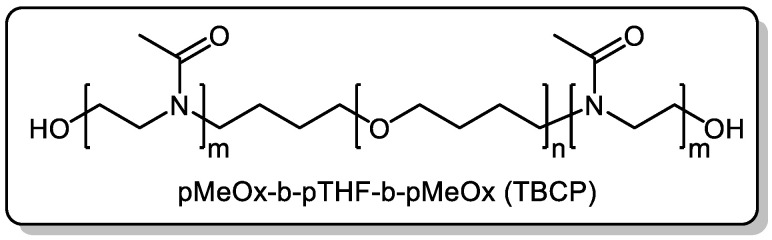
The chemical structure of TBCP triblock copolymer.

In 2020, Rasolonjatovo et al. prepared pMeOx-b-pTHF-b-pMeOx structure (TBCPs, Table 1) triblock copolymers to improve the efficiency of gene delivery to skeletal muscles [20]. Among them, TBCP1 (Figure 7; n = 7, m = 6) with the best effect of 1 mg/mL delivered pDNA encoding Luc to the TA muscles of mice. The expression of Luc in TBCP1 was eight times that of naked plasmid and two times that of 30 mg/mL Lutrol.

(2) Diblock copolymers

**Figure 8:**
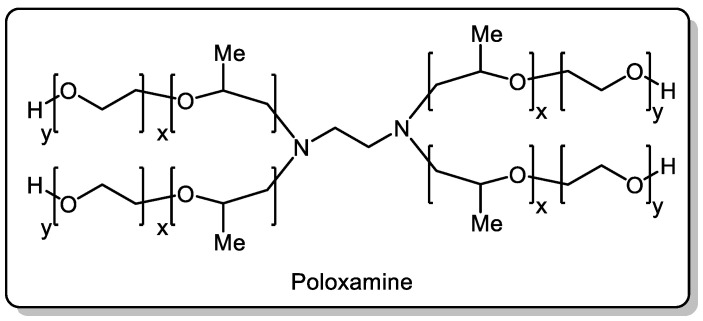
The chemical structure of Poloxamine.

In 2005, Richard et al. reported that they used PO_30_-EO_75_ composed of diblock copolymer Lutrol (Table 1), and Poloxamine 304 (x = 4, y = 4; Figure 8, Table 1) to study gene delivery [21]. pCMV-Luc and 0.3–3% (*w*/*v*) Lutrol were injected into the TA muscles of mice. Compared with the naked plasmid, the expression of luciferase increased 28 folds, and the equivalent delivery efficiency was obtained using 5% Poloxamine 304. The authors also used 0.3% Lutrol to deliver the pDNA encoding truncated dystrophin. No expression was found after injection of naked pDNA, but after injection of the mixed solution of Lutrol and pDNA, a stronger expression of dystrophin was found in the muscle surrounding fibers at the injection site on tissue sections, which indicated the effectiveness of Lutrol for gene delivery.

In 2006, Jang et al. reported that plasmid-loaded microspheres could provide local and sustained release of pDNA into target tissues, so it is possible to improve the efficiency of naked pDNA to promote transgene expression [22]. In this report, the microsphere design parameters were studied by correlating the degree and duration of transgene expression in the muscle with the molecular weight of the polymer and the quality of delivered DNA. Plasmid DNA was incorporated into poly(D, L-lactide-*co*-glycolide, Table 1) microspheres by low temperature double lotion method, and the microspheres were injected intramuscularly. The bolus injection of naked plasmid was used as a control, which showed the delivery of muscle cells whose transgene expression gradually decreased with time. The gene expression level in microspheres groups made of low molecular weight polymers increased from day 1 to day 92, and then decreased on day 174. Reducing the delivered microsphere quality resulted in simultaneous expression stabilization. However, the microspheres made of high molecular weight polymers were only expressed for 14 days. Understanding of the characteristics of microspheres that determine the expression of transgenes and the distribution of transferred cells may contribute to their application in tissue engineering or DNA vaccines.

In 2009, Basarkar et al. reported the use of poly(lactide-co-glycolide)-polymethacrylate (PLGA/E100, Table 1) diblock copolymer nanoparticles to deliver the plasmid encoding interleukin-10 (IL-10) into the muscle to prevent autoimmune diabetes in mice [23]. When the mixed solution of PLGA/E100 and the plasmid encoding IL-10 was injected into the TA muscles of diabetes model mice, the blood glucose concentration of PLGA/E100 group was similar to that of control untreated mice (normal mice) at all time points.

#### 3.1.2. Homopolymers

(1) PEI derivatives

When cationic polymers such as PEI were used to deliver genes, the cationic materials will strongly interact with serum, thus suppressing gene delivery and expression. As heparin has a shielding effect, combining heparin with cationic materials may improve the gene delivery efficiency in vivo. In 2008, Jeon et al. reported they combined heparin with branched polyethyleneimine (bPEI; 1800 Da) to form heparin-polyethyleneimine (HCPEI, Table 1) and used HCPEI for gene delivery [24]. The authors used pDNA encoding VEGF, and PEI (25 kDa), Lipofectamine, and bPEI (1800 Da) were used in positive controls. The complex of pDNA and polymer was injected into the skeletal muscle of the hind limb of mice. Compared with VEGF gene delivery using PEI (25 kDa), bPEI (1800 Da) or Lipofectamine, gene delivery using HCPEI was found to have significantly higher VEGF expression and wider neovascularization. HCPEI had lower cytotoxicity and higher blood biocompatibility than PEI (25 kDa) or Lipofectamine in vitro, showing a certain potential for gene delivery.

**Figure 9:**
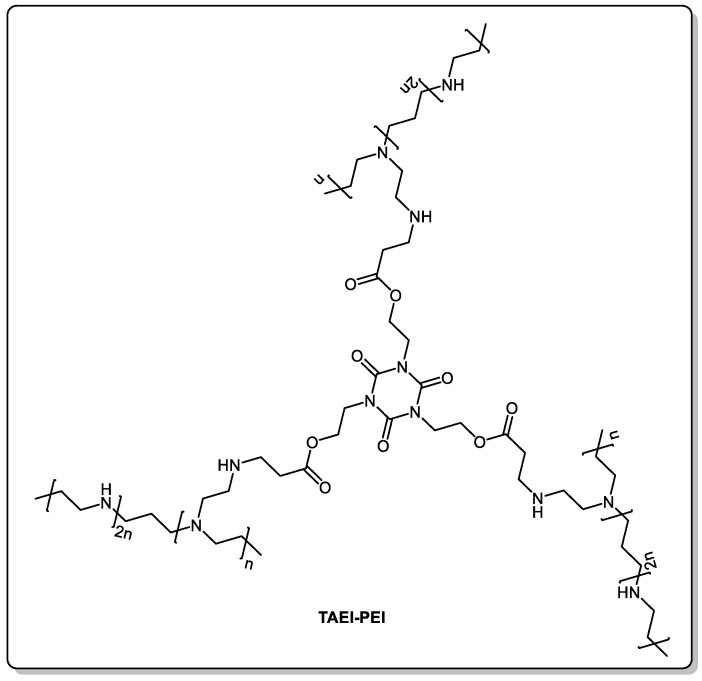
The chemical structure of TAEI-PEI.

In 2012, Wang et al. reported that hyperbranched poly(ester amine)s TAEI-PEI (Figure 9, Table 1) was successfully synthesized by Michael addition reaction between tris[2-(acryloyloxy)ethyl] isocyanurate (TAEI) and low-molecular-weight polyethylenimine (LPEI, Mw 0.8 k, 1.2 k, and 2.0) [25]. Compared with 25 kDa bPEI, the cytotoxicity of TAEI-PEI in two different cell lines was significantly reduced. By intramuscular administration, the gene expression of C12 (TAEI/PEI-2K at a ratio of 1:2) and C14 (TAEI/PEI-2K at a ratio of 1:4) in MDX mice were 5–8 times higher than that of 25 kDa bPEI. No obvious muscle damage was observed with the new polymer. The higher delivery efficiency and lower toxicity indicated the potential of biodegradable TAEI-PEI as safe and effective vectors.

(2) Other homopolymers

In 1996, Russell et al. studied materials for delivering genes to skeletal muscles [7]. At that time, the author selected polyvinylpyrrolidone (PVP, 50 kDa) and polyvinyl alcohol (PVA) for experimental research. The author first used chloramphenicol acetyltransferase (pCMV-CAT) as a reporter gene, and by using PVP (50 kDa) as a carrier, different concentrations of PVP (50 kDa) and pDNA were injected into the TA muscles of rats. It was found that CAT expression was highest when the concentration of PVP (50 kDa) was 5%. Then the authors used pCMV-β-gal as a reporter gene. The β-galactosidase expression of 5% PVP (50 kDa) was twice that of 10% PVP (10 kDa) and six times that of naked pDNA. When using PVA as a carrier to deliver pCMV-β-Gal, the β-galactosidase expression of 2% PVA (18 kDa) was about 10 times that of naked pDNA. It showed that polymers could effectively deliver genes. This work is one of the earliest studies on the skeletal muscle gene delivery materials, which pioneers the use of polymers for gene delivery to skeletal muscles.

In 2008, Kang et al. reported that gene delivery mediated by polymers could maintain the duration of pDNA administration [26]. In the study, PLGA (Table 1) polymers were evaluated as gene carriers. PLGA polymer loaded with pDNA had high encapsulation efficiency (87%). The polymers could release pDNA for 11 days. The released pDNA maintained its structural and functional integrity. In addition, PLGA polymer showed lower cytotoxicity than bPEI (25 kDa) in vitro and in vivo. The mixed solution of pDNA encoding VEGF and PLGA polymer was injected into the skeletal muscles of ischemic limbs of model mice and compared with PEI/pDNA or naked pDNA in vivo. After 12 days, PLGA polymer/pDNA had significantly higher VEGF expression levels than that of PEI/pDNA and naked pDNA. In addition, gene therapy using PLGA polymer resulted in more extensive neovascularization at the ischemic site compared with bare pDNA and PEI/pDNA. These results indicated that PLGA polymers could be used as potential carriers for gene delivery in skeletal muscles.

#### 3.1.3. Dendrimers

As a non-viral gene delivery vector, poly (amidoamine) (PAMAM) dendrimers have attracted great interest because of their high efficiency in gene delivery in vitro [70,71]. These dendrimers carried primary amine groups on their branched surfaces, which could bind and compress DNA into complexes, and promote the uptake of DNAs by cells [72]. Therefore, PAMAM dendrimers showed a high level of gene expression in a variety of cultured cells, especially PAMAM G5 (trade name SuperFect) [73]. As they could interact with negatively charged cell surfaces [74], cationic PAMAM dendrimers had certain cytotoxicity, hemolysis, and hepatotoxicity [75,76], which limited their wide applications in vitro and in vivo [77,78,79].

In 2009, Qi et al. reported that they used PEG-modified PAMAM for gene delivery research in skeletal muscles [3]. They first prepared a series of PEGylated PAMAM with different coupling degrees by chemical coupling PEG (MW. 5000 Da) on PAMAM G5 or G6. The PEG modification decreased the cytotoxicity and significantly increased the in vitro transfection efficiency. By intramuscular injection of PEG-G5/pDNA or PEG-G6/pDNA into the vicinity of the quadriceps muscle, strong expression of EGFP was found. Compared with naked pDNA, all PEG-G5 and PEG-G6 products could significantly enhance (usually more than several orders of magnitude) the expression of EGFP. Consistent with the results in cell culture, G5-8%PEG (8% showed PEG coupling degree) resulted in the highest expression of EGFP among all PEG-PAMAM (Table 1) dendrimers. In addition to G5-8%PEG, the second effective PEG-PAMAM was G6-8%PEG, which produced higher potency than other PEG-PAMAM.

In 2021, Hersh et al. modified the G5 PAMAM nanocarrier with a skeletal muscle-targeting peptide (SMTP), a DLC8-binding peptide (DBP) for intracellular transport, and a NLS peptide for nuclear uptake, and then polyplexed with pDNA [27]. The nanocarrier had less cytotoxicity. In in vitro experiments, nanoparticles with the optimal PAMAM-G5/SMTP/DBP/NLS/DNA (Table 1) ratio were used to transfect HEK 293T cells, and the transfection efficiency was lower than that of the commercial reagent Lipofectamine 2000. However, in C2C12 cells, the transfection efficiency of nanoparticles with the optimal PAMAM-G5/SMTP/DBP/NLS/DNA ratio was higher than that of Lipofectamine 2000. When micro-dystrophin was delivered to mice in vivo, the related proteins could also be successfully expressed in skeletal muscles.

#### 3.1.4. Peptides

In 2010, Itaka et al. reported the study of gene delivery to skeletal muscles of mice using PEG-poly(L-lysine) diblock co-polymers composed of PEG and poly(L-lysine) [43]. They used PEG-Poly(L-lysine) and pDNA to form polyplex nanomicelles by self-assembly. On the third day, compared with the naked plasmid group, the fluorescence of the nanomicelle group increased by 10 times, and reached the peak on the sixth day. Then they used the pDNA encoding a soluble form of VEGF receptor-1 (sFlt-1) to inhibit the growth of blood vessels in the process of tumour growth, so as to achieve the purpose of inhibiting tumours. The administration of PEG-Poly(L-lysine)/psFlt-1 significantly inhibited tumour growth compared with naked plasmid.

In 2012, Osada et al. reported the application of PEG-poly(L-lysine) diblock co-polymers composed of PEG (MW. 12,000 Da) and poly(L-lysine) for gene delivery to mouse skeletal muscle [80]. In experiments in vivo, they transferred naked pDNA targeting skeletal muscle tissue by intravenous injection combined with a tourniquet, which was placed on the proximal thigh prior to injection and kept for 5 min after injection to temporarily restrict blood flow. Using this method, pDNA-Luc mixed with multimeric micelles PEG-Plys was injected into the great saphenous vein of the distal hindlimb and exhibited significantly higher luciferase expression than that of naked pDNA.

In the process of non-viral vector-mediated gene delivery, entering the nucleus is the most difficult step. Only about 1% of pDNA can be internalized into cells and transferred to the nucleus. At the same time, it was also proved that at least 10^6^ plasmids were needed to be injected to observe the gene expression in TA muscle myotubes in mice [81]. In response, Lavigne et al. designed a chimeric system, which contained cell penetrating peptide TAT, a multi subunit DNA binding protein (M2S), and a plasmid encoding the α-A of galac-tosidase A (AGA). The chimeric system was used for the treatment of Anderson Fabry disease caused by the lack of AGA [82]. The results showed that this chimeric system increased the expression of therapeutic AGA gene by about eight times compared with the naked plasmid, and about four times compared with Pluronic^®^ SP1017. With the help of M_2_S-TAT, the expression of GFP was also increased by more than 10 times. The study indicated that binding of M_2_S-TAT to the plasmid could help the plasmid enter the cell nucleus, resulting in improved gene expression.

Laminin is the main component of the basement membrane. It can interact with α-dystroglycan (α-DG), an extracellular protein widely distributed on the surface of skeletal muscle cells. Suzuki and Negishi et al. screened several synthetic laminin peptides and identified an α-DG binding peptide, A2G80 (amino acid sequence VQLRNGFPYFSY) [83,84]. A2G80 peptide had high affinity to the α-DG [85]. Therefore, the authors designed the A2G80-R9 peptide to facilitate gene delivery into skeletal muscle cells. Santos et al. found that quinoline could help DNA escape from endosomes by preventing the decrease of pH in endosomes [86,87]. Therefore, Sasaki et al. used different concentrations of quinoline to mix with A2G80-R9-pDNA complex and found that quinoline could promote the pDNA transfer efficiency [88]. In addition, the author added an oligo histidine (H8) to A2G80 to form A2G80-R9-H8 peptide. The transfer efficiency of A2G80-R9-H8 peptide was independent of the concentration of quinoline. Luciferase and desmin proteins were found to co-localize in TA muscles, proving that A2G80-R9-H8 could enrich pDNA in muscle cells without non-targeted delivery.

### 3.2. Liposomes

In 1964, Bangham et al. observed that phospholipid molecules could spontaneously form a closed bilayer structure in water [89]. Since then, liposomes have become one of the most promising drug carriers. Liposomes have many advantages, such as low toxicity, high loading capacity, and good biocompatibility [90,91,92]. Liposomes were easy to fuse with each other, resulting in wide particle size distribution and ineffective drug release [93]. In subsequent studies, it was found that the problem of wide particle size distribution and effective drug release of liposomes could be improved by modifying liposomes with polyethylene glycol (PEG) [94], and the circulation time of liposomes in vivo could also be increased. At the same time, adding antibodies to the surface of liposomes (immunoliposomes) [95], using cationic lipid molecules (cationic liposomes) [96], modifying peptide chains on the surface (peptide-targeted liposomes) [97], using the specific environment of target tissues, preparing temperature sensitive liposomes [90,98] or pH sensitive liposomes [99], etc. could also make liposomes have targeting properties [94].

Trived et al. used four kinds of liposomes: LipofectAMINE [2,3-dioleyloxy-n-[2-(sperminecarboxamido) ethyl]-N, N-dimethyl-l-propidium trifluoroacetate:dioleoylphosph-atidylethanolamine (DOPE) = 3:1 (*wt*/*wt*)], Lipofectin [(n[1-(2,3-dioleyloxy) proxy]-N, N-trimethylamine chloride:DOPE = 1:1 (*wt*/*wt*)], LipofectACE [dimethyl dioctadecylammonium bromide and DOPE = 1:1.25 (*wt*/*wt*)], and N-[1-(2,3-dioleoloxy) proxy]-N, N, N-trimethylamonium methyl sulfate (DOTAP), to form a complex with the plasmid encoding dystrophin. Then the pDNA was transfected into C2C12 cells. After the C2C12 cells expressed dystrophins, they were implanted into the muscle tissues of mice, thus achieving the effect of gene therapy. Lipofectamine was the best with the gene transfection efficiency of 40% (DNA: Lipid = 1:5). The second was DOTAP, with a maximum efficiency of about 15–20% (DNA: lipid = 1:1) [100].

Although cationic liposomes have great application potential in gene transfection, there are many negatively charged proteins or polysaccharides in the ECM of muscle cells in animals. Both cationic liposomes and cationic polymers will be hindered by ECM when delivering genes to cells, thereby limiting the fluidity of the cationic carrier/pDNA complex in the tissue and reducing the delivery efficiency.

In 2018, Joan K. Ho et al. used self-crosslinked cationic lipopeptide (LP)-stearoyl Cys-His-Lys-Lys-Lys-amide (stearoyl CH_2_K_3_) for DNA delivery. Its principle was similar to that of PEI for gene delivery. First, the lipopeptide formed a complex with pDNA to protect DNA. After entering the target cells through endocytosis, the lipopeotide/pDNA complex escaped from the endosomes through the “proton sponge” effect [52]. Due to the positive charge of the material, it had certain cytotoxicity. In order to reduce cytotoxicity, the authors added 1,2-distearoyl-sn-glycoro-3-phosphoe-thanolamine-N-[methoxy (polyethylene glycol)-2000] (DSPE-PEG2000) to the cationic lipopeptide/DNA complex to form a PEGylated cationic lipopeptide/DNA complex. PEG can be used as a charge shielding agent in the preparation of cationic liposomes to enhance the stability of cationic liposome/DNA complex in human body and to improve its distribution, because PEG has the function of shielding the surface charge of cationic complexes. The results showed that after PEGylation, the surface charge of the cationic LP/DNA complex was reduced, thus decreasing the non-specific electrostatic effect of the complex in the extracellular components and preventing salt induced aggregation. The PEGylation improved the distribution of the complex in vivo, making it more absorbable by muscle cells, thus increasing the expression level of foreign genes. Moreover, DSPE-PEG2000/LP/DNA particles could be assembled in aqueous solutions without using organic solvents, which had certain manufacturing advantages [101].

## 4. Gene Delivery Methods to Skeletal Muscles

### 4.1. Electroporation

#### 4.1.1. Pluronic^®^ L64-Based Methods

In 2014, our research group reported that gene delivery to the skeletal muscle of mice could be further improved by combining Pluronic^®^ L64 with low-voltage electroporation [102]. The gene expression level was improved by 80 times compared with naked plasmid injection, and improved by 11 times as compared with gene delivery using Pluronic^®^ L64 or electroporation alone. The method is relatively mild and safe enough for clinical application.

However, in previous Pluronic^®^ L64-mediated intramuscular gene delivery methods, pDNAs were not compressed and kept loose structures, making them unstable in vivo and inefficient in the transmembrane process. In 2019, we screened out the *Epigallocatechin gallate* (EGCG), a natural compound in green teas, which could compress pDNAs to nanoscale [15]. By combining the EGCG-compressed pDNA and L64-electropulse (L/E) technique [102], we constructed the L/E/G intramuscular gene delivery system. Herein, L refers to 0.1% (*w*/*v*) Pluronic L64, E = electropulse, and G = EGCG. In the L/E/G system, the highest luciferase expression level increased to 7.6 times in comparison with that in the L/E group, and 122 times with pDNA injection alone. The study revealed that a key principle for material/pDNA complex benefitting intramuscular gene delivery was to get a negatively charged complex. Meanwhile, this proof-of-concept study provided a proper strategy to design more efficient biomaterials for intramuscular gene delivery and expression. As the L/E/G system is strong enough to produce functional proteins in vivo, we applied it for cancer immunotherapy [1] and type 1 diabetes treatment [2].

#### 4.1.2. Other Electroporation

For negatively charged pDNA, electroporation may produce higher delivery efficiency, because electroporation can not only produce pores on the cell membrane, but also push pDNA easier to enter the cell membrane driven by the electric field force, and even promote DNA nucleation under the action of the electric field force. However, too high an electric field will damage the tissue. In addition, the transfer area is only between two electrodes, making it unable to transfer a large area. Kusumanto et al. compared the electroporation and ultrasound microbubble methods, and found that the average positive staining area of electroporation was 1.80 mm^2^, and the average positive staining area of ultrasound microbubble method was 0.05 mm^2^. It can be seen that electroporation has better transfer results [103], but at the same time, electroporation may also produce unexpected damage on external muscle fibers [104].

Electroporation also has certain limitations. If we want to achieve high gene delivery efficiency, we must use a high-intensity electric field, which may also lead to irreversible perforation on cell membranes, resulting in unexpected irreversible damage to the target tissues. At same time, under a high-intensity electric field, there will also be an electric current since ions exist in the cytoplasm and ECM. The enhancement of the current will also enhance the thermal effects and continue to cause irreversible damages to the target tissues. This is precisely the biggest limitation of the electroporation.

### 4.2. Methods Combining Microbubble and Ultrasound

Unger et al. found that ultrasound-mediated microbubble technology could improve the efficiency of gene delivery into tissues [105]. The skeletal muscle is one of the ideal targets for ultrasound and microbubble mediated gene delivery. The diameter of the microbubbles is about 3 μm. It is mainly used as a contrast agent and can improve ultrasound scanning. The principle of ultrasonic gene delivery is to create transient non-lethal membrane pores on the cell membrane, thereby increasing the transmembrane success rate of pDNA on the cell membrane. Microbubbles can generate shock waves through the cavitation effect at low ultrasonic power, generating membrane pores on the surface of cell membranes and reducing unexpected damage to target tissues [106].

In 2012, Burke et al. combined PEI-PEG with ultrasound microbubbles and delivered plasmid encoding luciferase to the skeletal muscles of C57BL/6 mice. Under ultrasound conditions, the intensity of bioluminescence imaging was about 180 times more intense than that without ultrasound. When the peak sound pressure of ultrasound was 0.6 MPa, the muscle luciferase activity was significantly improved. The muscle luciferase activity reached the peak on the seventh day. During gene delivery, no obvious signs of necrosis, regeneration or leukocyte infiltration was found in the muscles [107].

Chen et al. combined microbubbles with Pluronic^®^ P85 and delivered the pDNA encoding GFP using an ultrasound-assisted method. They found that the number of GFP positive fibers in the mouse skeletal muscles was increased by about three times in comparison with ultrasound-assisted microbubble delivery alone. Certain muscle damages were caused in the process of ultrasound or near the injection needle hole. If there was no ultrasound, there was no damage [108].

Although ultrasound can achieve relatively good delivery efficiency at low energy, few literatures were found to study the influence of factors such as contrast agent microbubbles, sound pressure, and different doses of pDNA on the efficiency of ultrasound-mediated gene delivery. It can be inferred from previous studies that there should be an optimal set of parameters to make the gene delivery efficiency the highest. Shapiro et al. studied the skeletal muscles of mice and found that the sound pressure was 200 kPa and the microbubbles were 5 × 10^5^ bubbles. When the ultrasonic treatment time was 2 min and the amount of pLuc2 (Luciferase 2) was 50 μg/site, the expression of luciferase reached the largest. From the fourth day to the seventh day after treatment, the expression of luciferase was much higher than those in other groups. When ultrasound microbubbles acted synergistically, the expression of luciferase was higher than that of the group without synergism [109].

In addition to the parameters of ultrasound, the characteristics of microbubbles may also affect the transfection efficiency of DNA [110]. Panje et al. used cationic microbubbles and neutral microbubbles with the same diameter to compare them respectively. First, when pDNA and cationic microbubbles had better binding, cationic microbubbles had better protective effects on pDNA. Later, pFluc was used to transfect vascular endothelial cells. The expression of cationic microbubbles group was 14.5 times higher than that of neutral microbubbles group. When 50 μg of pFluc and 1 × 10^8^ cationic microbubbles were transferred into the TA muscles of mice, it was found that the signal intensity was the highest at 120 h after administration, and the signal of cationic microbubbles was stronger than that of neutral microbubbles. Before reaching 1 × 10^8^ microbubbles, the more the number of microbubbles, the higher the transfer efficiency, but after that point, the transfer efficiency was hardly increased [111].

Both liposomes and ultrasound microbubbles have the potential for gene delivery. If they are combined, they may produce better effects. In 2012, Negishi et al. combined liposomes with DPPC and PEG2000 in a molar ratio of 94:6, mixed them with contrast agent microbubbles to prepare foam liposome BLs, and then loaded FITC labelled pDNA to make p-BLs. They studied the effects of various factors, such as the presence of DOTAP lipids and the ratio of PEG2000 to PEG750. They found that liposomes containing DOTAP could improve ultrasound imaging, and the ultrasound signal was stronger than that of BLs containing only DPPC. Moreover, it could capture imaging gas more effectively, but the loading of pDNA was slightly reduced. At the same time, more PEG750 could improve the loading of pDNA, but too few PEG2000 molecules would also affect the stability of BLs in vivo. Finally, the best ration of PEG2000:PEG750 was 2:4. In serum environment, BLs could protect pDNA from being degraded. In experiments in vivo, p-BLs loaded with basic fibroblast growth factor (bFGF) achieved efficient treatment of hindlimb ischemia in mice, increased the mRNA quantity of angiogenic genes, and improved the hindlimb blood flow [112]. When the ultrasonic intensity was about 2 W/cm^2^, there was no obvious damage on the TA muscles of mice, but at 4 W/cm^2^, tissue damage was found, indicating that the ultrasonic intensity should not exceed 4 W/cm^2^, and there should be a suitable range to ensure the highest gene transfer efficiency and ensure biological safety [113]. In order to further explore whether other cationic lipids could promote the delivery of ultrasound foam liposomes, Negishi et al. prepared and studied four kinds of cationic lipids— DOTAP, 1,2-stearoyl-3-trimethylamonium-propane (DSTAP), 1,2-distearoyl-3-dimethy-lamonium-propane (DSDAP), dimethyloctadecylammonium bromide (DDAB)—mixed with contrast microbubbles to prepare four kinds of foam liposomes (BLs). Among them, liposomes containing DSDAP showed the best performance. The authors studied the signal strength of these cationic liposomes as ultrasound contrast agents and found that the liposomes containing DSDAP had the highest signal strength, even higher than of the foam liposomes with DOTAP [112], which had been reported to have the application potential while combined with ultrasound. In gene delivery experiments on the mouse skeletal muscles, pDNA and siRNA were loaded in the foam liposomes to generate p-BLs and si-BLs [112], and then combined them with an ultrasound imaging system to treat mouse hindlimb ischemia. The results showed that in the presence of ultrasound, liposomes containing DSDAP showed the highest gene delivery efficiency. Moreover, BLs containing DSDAP were not only more stable, but also have the largest loading capacity of pDNA. These four BLs could also protect the pDNA from degradation in serum [107,114].

### 4.3. Gene Gun

It deposits pDNAs on gold particles, and then accelerates the gold particles by pressurized gas to make them penetrate cell membranes with high kinetic energy, and delivers pDNAs into target cells [47]. pDNAs can even be directly delivered into the nucleus. After bombarding skeletal muscles, the gene delivery efficiency to muscle cells was 1–5%, and the penetration depth of particles was not more than 0.5 mm, but the gene expression time can reach 60 days [115].

## 5. Conclusions and Prospect

For non-viral material-mediated gene delivery to skeletal muscles, each material and method has its own advantages and disadvantages. For liposomes and polymers, which are like skeletons, different modification groups or surface modifications can be added to them according to different requirements, so they have a variety of functions to complete gene delivery. Therefore, polymers and liposomes as non-virus gene delivery materials have broad research prospects. Moreover, non-viral gene delivery materials also tend to be applied in complex ways, such as cationic lipopeptide [101], foam liposome BLs made of ultrasound contrast agent microbubbles mixed with traditional liposomes [114], the combination of Pluronics with ultrasound microbubble [108], and the addition of A2G80-R9 peptide to functional octamer histidine to form A2G80-R9-H8 which had enhanced endosome escape ability [116]. Moreover, the recombination of these gene delivery methods may also greatly enhance the delivery efficiency of foreign genes. For different gene delivery systems, studies have also proven that reasonable experimental parameters could also improve gene delivery efficiency and expression level [107].

Therefore, combining different delivery methods and screening optimal experimental parameters may produce higher delivery and expression results with less side efforts. It is believed that with the recombination and advancement of the skeletal muscle gene delivery technology, the target gene expression level will be gradually improved, so that more diseases can be therapied through this simple, safe, convenient, economical, and efficient technology platform for gene therapy.

## Figures and Tables

**Table 1:** Polymers for gene delivery to skeletal muscles.

Polymers	Year	Reporter Genes	Functional Genes	Function
Polyvinyl pyrrolidone (PVP)	1996 [7]	CAT, β-CAT	/	Detecting gene expression
Pluronic^®^ P85	2005 [8]	Luc	/	Detecting gene expression
	2009 [9]	GFP, Luc	/	Detecting gene expression
	2016 [10]	Luc, GFP	pDRIVE5Lucia-mDesmin & pDRIVE5GFP-mDesmin	Treating hind limb ischemia in mice
Multi-block copolymers from Pluronic^®^ P85 and di-(ethylene glycol) divinyl ether	2009 [11]	Luc	/	Detecting gene expression
Pluronic^®^ SP1017	2005 [8]	Luc	/	Detecting gene expression
Pluronic^®^ 25R2	2011 [12]	Luc	/	Detecting gene expression
Pluronic^®^ L64	2002 [13]	Luc, β-Gal	/	Detecting gene expression
	2014 [14]	/	HIF-1α	Treating severe hind limb ischemia in mice
	2019 [15]	lacZ, Luc, E2-Crimson	GHRH	Detecting gene expression Accelerating mouse growth
	2021 [1]	E2-Crimson, EGFP	mlFVII Icon	Detecting gene expression Tumor Immunotherapy
PEG_13_-PLGA_10_-PEG_13_	2007 [16]	Luc	VEGF	Detecting gene expression
PEO–PTHF–PEO	2008 [17]	Luc, lacZ	/	Detecting gene expression
LPL	2014 [18]	lacZ, Luc, E2-Crimson	mGH	Detecting gene expression Accelerating mouse growth
rL_2_PL_2_, rL_3_PL_3_	2016 [19]	lacZ, Luc, E2-Crimson	GHRH	Detecting gene expression Accelerating mouse growth
TBCPs	2020 [20]	Luc	/	Detecting gene expression
Lutrol	2005 [21]	Luc	/	Detecting gene expression
Poloxamine 304				
poly(D, L-lactide-*co*-glycolide)	2006 [22]	Luc	/	Detecting gene expression
PLGA/E100	2009 [23]	/	IL-10	Treating type 2 diabetes
HCPEI	2008 [24]	/	VEGF	Treating mouse ischemic limb
TAEI-PEI	2012 [25]	GFP	/	Detecting gene expression
PLGA	2008 [26]	/	VEGF	Treating mouse ischemic limb
PEG-PAMAM	2009 [3]	EGFP	/	Detecting gene expression
PAMAM-G5/SMTP/DBP/NLS mixture	2021 [27]	EGFP	micro-dystrophin (µDys)	Detecting gene expression Treating Duchenne Muscular Dystrophy

## Data Availability

Not applicable.
